# Transient ischemic attack presenting as recurrent migratory numbness by seconds: a rare case confirmed by transcranial Doppler micro-emboli monitoring

**DOI:** 10.1186/s12883-020-01955-2

**Published:** 2020-11-23

**Authors:** Xianyue Liu, Ke Han, Mingyi Hu, Huanquan Liao, Qinghua Hou

**Affiliations:** The Clinical Neuroscience Center, The Seventh Affiliated Hospital, Sun Yat-sen University, Shenzhen, 518107 China

**Keywords:** Transient ischemic attack, Transcranial Doppler, Micro-embolic signals, Migratory numbness, Case report

## Abstract

**Background:**

Transient ischemic attack (TIA) is a brief episode of cerebral ischemia. However, if a symptom is not presented as drop attack or hemiplegia, and alarming to the patient and the physician, how short of a symptom duration would raise the concern of a physician for TIA? It will be more complicated if the location of the neurological deficit is vagrant. This report highlights a rare TIA case which presented a very short duration of migratory patchy distribution numbness.

**Case presentation:**

A middle-aged gentleman was presented with recurrent patchy distribution numbness on the right side of the body for 2 months, with the episode lasting as short as about 10 s. The location of the numbness was erratic and migratory. Magnetic resonance angiography (MRA) revealed mild stenosis on the left middle cerebral artery (MCA). Transcranial Doppler (TCD) micro-emboli monitoring detected positive micro-emboli signals (MES), leading to the confirmation of a TIA diagnosis. After a standard dual antiplatelet treatment combined with enhanced lipid reduction therapy with statins, MES disappeared on dynamic TCD emboli monitoring, and no more episodes of TIA have been noticed on the follow-ups.

**Conclusion:**

TIA caused by micro-emboli can display as recurrent migratory neurological deficit within seconds. TCD micro-emboli monitoring is very helpful to differentiate this situation from TIA mimics with follow-ups, as well as to locate unstable plague.

## Background

TIA is a transient episode of neurological dysfunction caused by focal brain, spinal cord, or retinal ischemia, without acute infarction [1]. The symptom of a typical TIA case will vanish most likely within 1 h [2], usually 5 to 15 mins [3], but rare in several seconds. If the symptom duration is as short as usually unconsidered seconds and the location of the neurological deficit is vagrant, the diagnosis of TIA is difficult to be established. We here report a rare case of TIA with repeated, migratory, rapidly-disappeared patchy distribution of numbness on one side of the body, which is confirmed by TCD micro-emboli monitoring.

## Case presentation

A 44-year-old man, accounting himself to be basically healthy except for smoking for more than 20 years, presented in the clinic. The major complaint of this gentleman for this visit is that he has continually experienced “recurrent numbness on the right side” in the past 2 months. The paroxysmal numbness is patchy, without weakness of the limbs, slurred speech and skewed mouth. The symptoms lasted only about 10 to 30 s. The frequency of the symptoms varied from once a week to 6 times a day. The distribution of the numbness is erratic, mostly within the upper extremity, followed by the distal part of the lower extremity, and the least on the trunk, but all on the right side of the body. Through careful medical history interrogation, we found out that 2 years ago, this gentleman had manifested a symptom of inflexible right hand during a Mahjong playing, which lasted for about 5 mins and resolved totally. A similar inflexible right-hand symptom had recurred 3 times in the most recent 2 months. Furthermore, this man noticed an elevated fasting blood glucose on a physical examination performed 8 months prior to admission, but he did not take any pertinent measures to improve it except for the recent weeks’ intake of metformin. These findings lead to the admission of this patient.

On admission, physical examination found no obvious abnormalities except for his obesity with a Body Mass Index (BMI) of 28.2 kg/m^2^. Lab investigation only found mild-elevated serum lipids (TC 5.20 mmol/L, TG 3.04 mmol/L, LDL-C 2.85 mmol/L) and hyperglycemia (a fasting blood glucose of 7.56 mmol/L and a HbA1C of 7.1%).

**Fig. 1:**
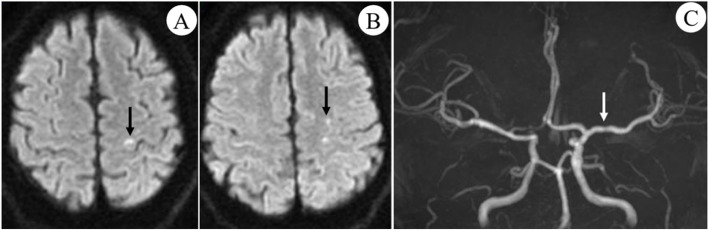
MRI-DWI and MRA findings. **a**, **b** multiple high signals on DWI located within the left prefrontal cortex and centrum semiovale (black arrows); **c** MRA showed mild focal stenosis on the LMCA horizontal segment (white arrow)

Auxiliary investigations showed that 24-hrs electrocardiogram (ECG), color echocardiography, electromyography (EMG) and electroencephalogram (EEG) were all normal. A single flat plaque was detected on the right subclavian artery on carotid ultrasound. MRI-Brain diffusion weighted imaging (DWI) indicated multiple spotted hyperintensities within the left prefrontal cortex and centrum semiovale (Fig. 1), suggesting recent multifocal diffusion-restricted lesion caused by artery-to-artery embolism (corresponding to the upper extremity area of the precentral gyrus, which is highly consistent with the 3 times of inflexible hand symptom that had happened within the past 2 months). MRA showed mild focal stenosis at the horizontal segment of left MCA (Fig. 1), which is quite un-conspicuous.

TCD showed that a much-increased blood flow velocity was detected at left MCA and left anterior cerebral artery (ACA) (142 cm/s and 147 cm/s, respectively) with distorted spectral waveforms, indicating mild focal stenosis at these two arteries.

**Fig. 2:**
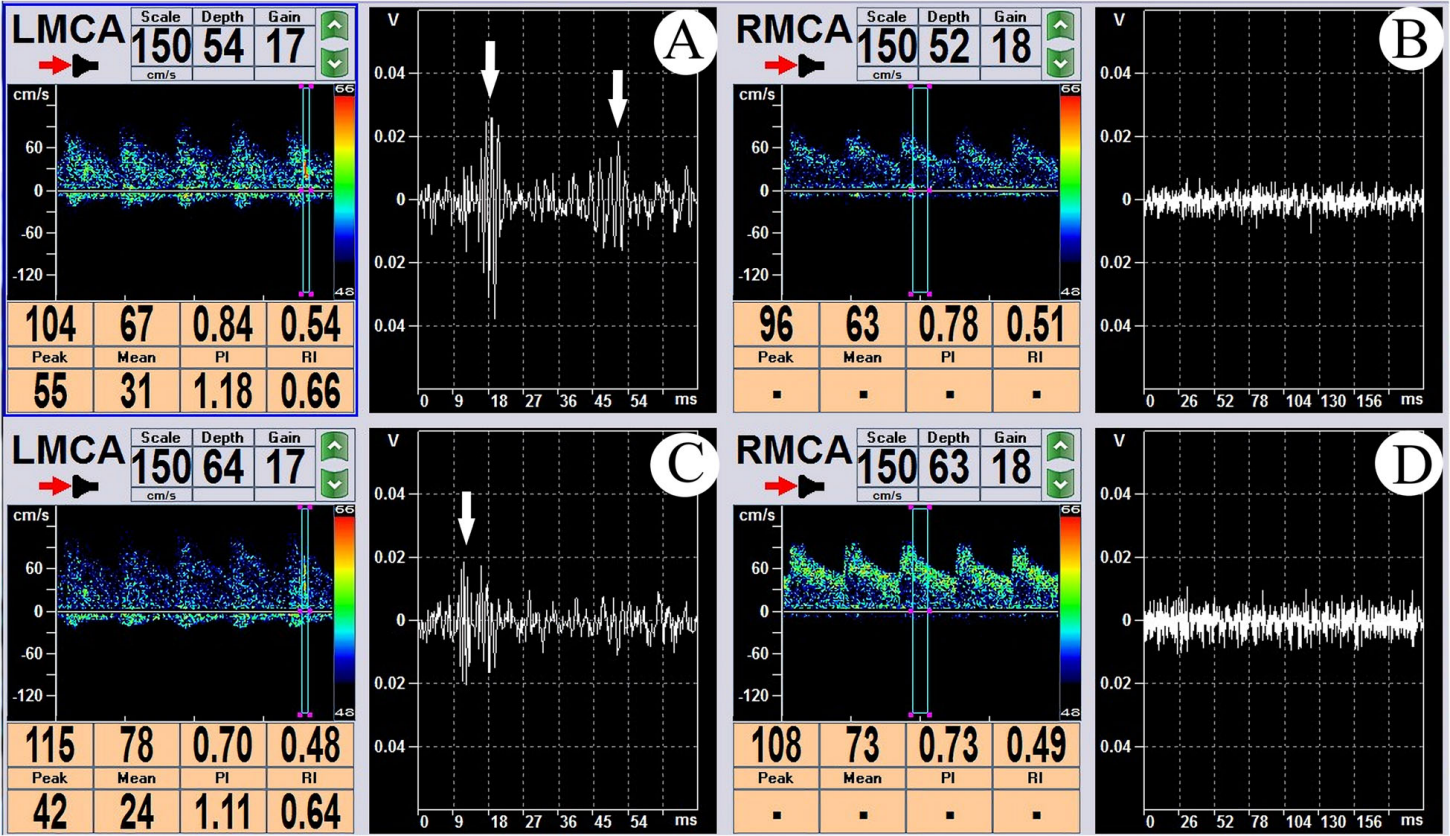
Dual-channel, dual-depth TCD emboli monitoring. **a, b** TCD emboli monitoring of LMCA at a depth of 54 cm (**a**) and 64 cm (**b**), white arrows indicating the MES. It could be seen that the emboli was first detected at the depth of 64 cm, and then moved toward the distal end of the blood vessel and was broken into two at the depth of 54 cm. **c, d** No MES were observed at RMCA either at the depth of 52 cm (**c**) or of at the depth of 63 cm (**d**)

A dual-channel, dual-depth TCD micro-emboli monitoring protocol was applied, and the sample volume was set as 10 mm all through. At a depth of 54-64 mm, 4 MES were detected at left MCA within a 30 mins time duration. At a depth of 52-63 mm at right MCA and a depth of 70 mm at left ACA, no MES were detected within 30 mins (Fig. 2). A double-check monitoring was conducted, and 6 MES were detected at left MCA within 30 mins. Again, no MES were detected at right MCA and left ACA.

The diagnosis was then established as clinical TIA with diffusion-restricted lesion and type 2 diabetes mellitus. Aspirin 100 mg q.d., clopidogrel 75 mg q.d., atorvastatin 40 mg q.n., and blood glucose control therapy were then given to the patient, followed by a series of TCD micro-emboli monitoring to evaluate the effect. After just 2 days of treatment, the number of detected MES decreased to 2 on the 30 mins continuous monitoring, and with zero at the serial TCD monitoring at 3 days, 5 days and 3 months after treatment. A follow-up of clinical symptoms lasting for 1 year was also negative.

## Discussion and conclusion

The diagnosis of TIA can be very challenging. In the evolutionary definition of TIA, though the duration limitation of symptoms of < 24 hrs remains, the advances of imaging techniques is continuously cutting short the time duration needed to cause an ischemic lesion and tend to identify more and more ischemic strokes from a clinically defined TIA [4]. However, in making the diagnosis of TIA other than a TIA mimic, the most could be relied on is still, with no doubt, clinical symptoms [5]. Although scoring indicators integrated with medical history, vascular events and risk factors have been developed to increase the accuracy of diagnosis of TIA [6], there is poor diagnostic agreement even among experts [7]. As for how long a symptom duration is long enough to raise the concern of the referred physician for TIA, no study has been conducted among neurologists, let alone among general practitioners. In a recent study, Counts et al. [8] pooled international multicenter cohort study of 1028 patients with lower risk transient or minor symptoms, and found out that 11.9% of patients had a symptom duration < 5 mins, suggesting a short duration of symptom is not that rare in clinical practice. But what if the symptom duration is as short as counting in seconds, especially when symptoms presented are not those classified as “higher risk”, such as dyskinesia or aphasia [9], like the patchy numbness manifested in the present case, and/or in an erratic migratory manner? And what if the symptoms have emerged less frequently than the present case? Will the chance be big that it will be neglected by general practitioners, and even by neurologists as well? This case has really enriched our knowledge of TIA.

**Fig. 3:**
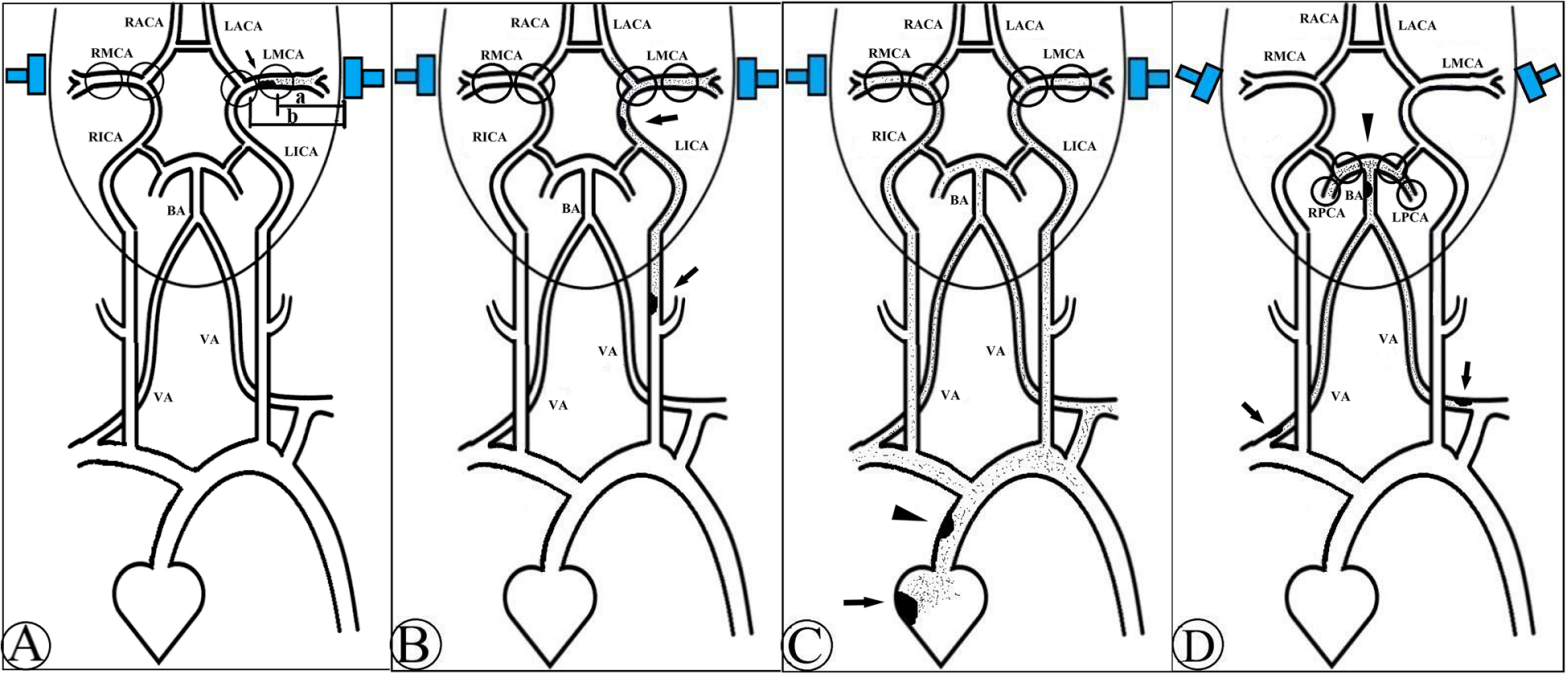
The ideograph of emboli sources investigated by TCD micro-emboli monitoring. **a** If MES are only detected on one side of MCA (like the left side as presented), and at the depth of a (MCA level), but not at the depth of b (ACA level), it then indicates that the embolus are originated from the MCA segment between a and b (black arrow indicates the embolus resource). **b** If MES are detected both at MCA and ACA on ipsilateral side, then the embolus are most likely derived from ICA of the same side (black arrows). **c** If MES are detected on both sides, then the embolus can be originated from the heart (black arrow), or ascending aorta (black arrow head). **d** If MES are detected at PCA but not MCA, it indicates that the embolus are originated from the posterior circulation, like BA (black arrow head), or VA (black arrows)

When a TIA is suspected, and the structural basis of hypoperfusion mechanism is lacking, it is then reasonable to turn to the micro-embolism mechanism. In the present case, the MES are detected only on the left side during the bilaterally monitoring, which indicates the possibility of cardiac original embolus is low. Furthermore, it can be determined that the emboli are originated from the horizontal section of left MCA rather than elsewhere, because MES are visible at a depth of 54–64 mm, but not at a depth of 70 mm where left ACA is branched from the internal carotid artery (ICA) terminal (for better illustrating how did we locate different embolic sources, we drew a series of ideograph of TCD micro-emboli monitoring as Fig. 3) [10]. This is further reconfirmed by the DWI-positive lesions presented only in the territory of left MCA, and the un-conspicuous mild stenosis of left MCA detected by MRA and TCD.

In summary, our case shows that TIA caused by micro-emboli can display as recurrent migratory neurological deficit by seconds, which deserves further attention. By using TCD emboli monitoring, we can not only differentiate this situation from TIA mimics, but also evaluate the effect of treatment, and what’s more, the location of unstable plaque that produces emboli can be determined. Of note, the failure to perform a high-resolution MRI analysis of the plaque composition of the criminal vessel wall is a drawback.

## Data Availability

All data related to this case report are contained within the manuscript and available from the corresponding author on reasonable request.

## Abbreviations

TIA: Transient ischemic attack
MRA: Magnetic resonance angiography
MRI: Magnetic resonance imaging
DWI: Diffusion weighted imaging
TCD: Transcranial Doppler
MES: Micro-emboli signals
BMI: Body Mass Index
TC: Total cholesterol
TG: Triglyceride
LDL-C: Low-density lipoprotein-cholesterol
HbA1C: Hemoglobin A1C
ICA: Internal carotid artery
VA: Vertebral artery
BA: Basilar artery
MCA: Middle cerebral artery
ACA: Anterior cerebral artery
PCA: Posterior cerebral artery
L: Left
R: Right

## References

[1] Easton JD, Saver JL, Albers GW, Alberts MJ, Chaturvedi S, Feldmann E, Hatsukami TS, Higashida RT, Johnston SC, Kidwell CS, Lutsep HL, Miller E, Sacco RL, American Heart Association; American Stroke Association Stroke Council; Council on Cardiovascular Surgery and Anesthesia; Council on Cardiovascular Radiology and Intervention; Council on Cardiovascular Nursing; Interdisciplinary Council on Peripheral Vascular Disease (2009). Definition and evaluation of transient ischemic attack: a scientific statement for healthcare professionals from the American Heart Association/American Stroke Association Stroke Council; Council on Cardiovascular Surgery and Anesthesia; Council on Cardiovascular Radiology and Intervention; Council on Cardiovascular Nursing; and the Interdisciplinary Council on Peripheral Vascular Disease. The American Academy of Neurology affirms the value of this statement as an educational tool for neurologists. Stroke.

[2] Albers GW, Caplan LR, Easton JD (2002). Transient ischemic attack-proposal for a new definition. N Engl J Med.

[3] Mohr JP (2014). History of transient ischemic attack definition. Front Neurol Neurosci.

[4] Wong KS, Caplan LR, Lim JS (2016). Stroke mechanisms. Front Neurol Neurosci.

[5] Fitzpatrick T, Gocan S, Wang CQ, Hamel C, Bourgoin A, Dowlatshahi D, Stotts G, Shamy M (2019). How do neurologists diagnose transient ischemic attack: a systemic review. Int J Stroke.

[6] Dolmans LS, Lebedeva ER, Veluponnar D, van Dijk EJ, Nederkoorn PJ, Hoes AW, Rutten FH, Olesen J, Kappelle LJ, MIND-TIA Study Group (2019). Diagnostic accuracy of the explicit diagnostic criteria for transient ischemic attack: a validation study. Stroke..

[7] Castle J, Mlynash M, Lee K, Caulfield AF, Wolfford C, Kemp S, Hamilton S, Albers GW, Olivot JM (2010). Agreement regarding diagnosis of transient ischemic attack fairly low among stroke-trained neurologists. Stroke..

[8] Coutts SB, Moreau F, Asdaghi N, Boulanger JM, Camden MC, Campbell BCV, Demchuk AM, Field TS, Goyal M, Krause M, Mandzia J, Menon BK, Mikulik R, Penn AM, Swartz RH, Hill MD. Diagnosis of uncertain-origin benign transient neurological symptoms (DOUBT) study group. Rate and prognosis of brain ischemia in patients with lower-risk transient or persistent minor neurologic events. JAMA Neurol. 2019. doi: 10.1001/jamaneurol.2019.3063. 10.1001/jamaneurol.2019.3063 PMC6763989 31545347

[9] Lodha N, Patel P, Harrell J, Casamento-Moran A, Zablocki V, Christou EA, Poisson SN (2019). Motor impairments in transient ischemic attack increase the odds of a positive diffusion-weighted imaging: a meta-analysis. Restor Neurol Neurosci.

[10] Gao S, Wong KS (2004). Transcranial Doppler (TCD) ultrasound diagnostic technique and clinical application.

