# Correction to: Transient ischemic attack presenting as recurrent migratory numbness by seconds: a rare case confirmed by transcranial Doppler micro-emboli monitoring

**DOI:** 10.1186/s12883-020-02026-2

**Published:** 2020-12-23

**Authors:** Xianyue Liu, Ke Han, Mingyi Hu, Huanquan Liao, Qinghua Hou

**Affiliations:** The Clinical Neuroscience Center, The Seventh Affiliated Hospital, Sun Yat-sen University, Shenzhen, 518107 China

**Correction to: BMC Neurol 20, 423 (2020)**

**https://doi.org/10.1186/s12883-020-01955-2**

Following publication of the original article [1], the authors reported an error in caption of Fig. 2. The correct caption is presented below.

**Fig. 2:**
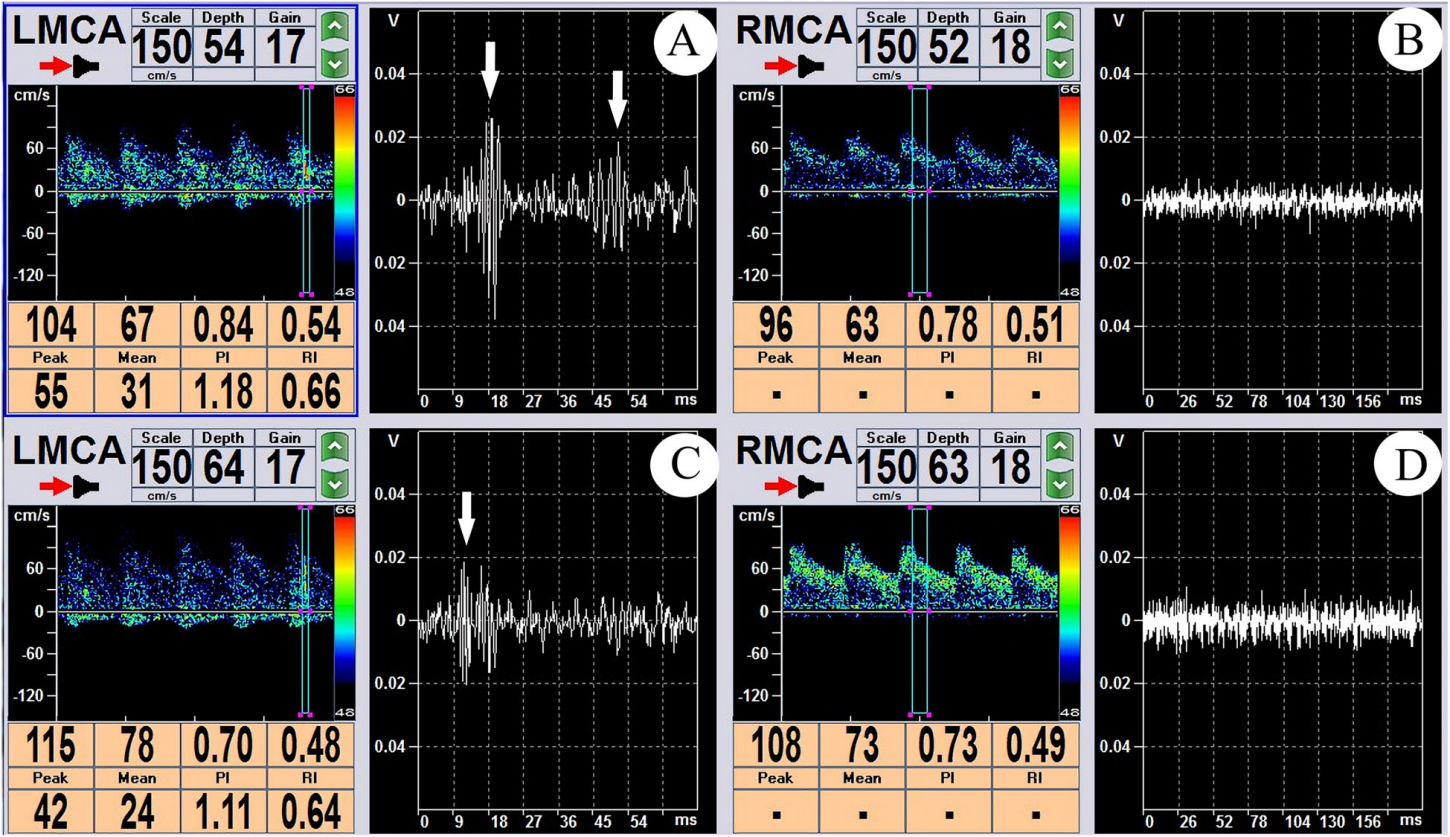
Dual-channel, dual-depth TCD emboli monitoring. **a**, **c** TCD emboli monitoring of LMCA at a depth of 54 cm (**a**) and 64 cm (**c**), white arrows indicating the MES. It could be seen that the emboli was first detected at the depth of 64 cm, and then moved toward the distal end of the blood vessel and was broken into two at the depth of 54 cm. b, d No MES were observed at RMCA either at the depth of 52 cm (**b**) or of at the depth of 63 cm (**d**)

The original article has been corrected.
